# ArfB can displace mRNA to rescue stalled ribosomes

**DOI:** 10.1038/s41467-020-19370-z

**Published:** 2020-11-03

**Authors:** Christine E. Carbone, Gabriel Demo, Rohini Madireddy, Egor Svidritskiy, Andrei A. Korostelev

**Affiliations:** 1 RNA Therapeutics Institute, Department of Biochemistry and Molecular Pharmacology, UMass Medical School, Worcester, Massachusetts 01605 United States; 2 Present Address: Central European Institute of Technology, Masaryk University, Kamenice 5, Brno, 625 00 Czech Republic; 3 Present Address: Medicago Inc., 7 Triangle drive, Durham, NC 27713 USA; 4 Present Address: Sanofi, 49 New York Ave, Suite 3660, Framingham, MA 01701 USA

**Keywords:** Ribosomal proteins, Ribosome, Structural biology

## Abstract

Ribosomes stalled during translation must be rescued to replenish the pool of translation-competent ribosomal subunits. Bacterial alternative rescue factor B (ArfB) releases nascent peptides from ribosomes stalled on mRNAs truncated at the A site, allowing ribosome recycling. Prior structural work revealed that ArfB recognizes such ribosomes by inserting its C-terminal α-helix into the vacant mRNA tunnel. In this work, we report that ArfB can efficiently recognize a wider range of mRNA substrates, including longer mRNAs that extend beyond the A-site codon. Single-particle cryo-EM unveils that ArfB employs two modes of function depending on the mRNA length. ArfB acts as a monomer to accommodate a shorter mRNA in the ribosomal A site. By contrast, longer mRNAs are displaced from the mRNA tunnel by more than 20 Å and are stabilized in the intersubunit space by dimeric ArfB. Uncovering distinct modes of ArfB function resolves conflicting biochemical and structural studies, and may lead to re-examination of other ribosome rescue pathways, whose functions depend on mRNA lengths.

## Abstract (web summary)

Alternative rescue factor B (ArfB) is an enzyme that releases peptides from stalled ribosomes to allow ribosome recycling. Here the authors carry-out cryo-EM analyses of 70S ribosomes complexed with ArfB on either a short or longer mRNA to reveal distinct modes of ArfB function.

## Introduction

A translating ribosome is normally recycled after the release of the nascent protein at the end of the mRNA open reading frame. Bifunctional proteins called release factors (RF1 and RF2 in bacteria) recognize stop codons and hydrolyze peptidyl-tRNA, discharging the peptide from the ribosome. The hydrolysis is catalyzed by the universally conserved GGQ motif of RFs in the peptidyl transferase center (PTC) of the ribosome (reviewed in refs. ^1–5^). However, ribosomes may stall on an mRNA due to rare codons, mRNA structure, or the absence of a stop codon caused by truncation or mutation^6,7^, giving rise to 2–4% of stalled ribosomes in *E. coli*^8^. Without a stop codon, various rescue strategies are used to release the peptides from stalled ribosomes^7,9^. Bacterial alternative rescue factor B (ArfB; formerly YaeJ) is a small ~140-aa peptidyl-tRNA hydrolase that lacks a stop-codon recognition domain. The GGQ-containing N domain is structurally similar to those of release factors, while the C-terminal tail is disordered in solution^10^. Eukaryotes encode a homologous protein ICT1 (“immature colon carcinoma transcript-1”, also termed MRPL58), essential in human cells^11,12^. ICT1 serves as a mitochondrial ribosome rescue factor^12,13^ and large-subunit mitochondrial ribosomal protein^14^. Crystal structure of a 70S•ArfB ribosome complex with an mRNA truncated at the P site codon (i.e., no overhang in the A site) revealed ArfB in the A site^15^. The C terminus is folded into an α-helix in the mRNA tunnel, whereas the catalytic N-terminal domain is inserted into the PTC. Positively-charged C-terminal residues are stabilized by interactions with the 16S ribosomal RNA nucleotides forming the mRNA tunnel, rendering the C-terminal tail the sensor of ribosomes with a vacant tunnel. While this position of ArfB is incompatible with a longer mRNA extending into the tunnel^15^, biochemical data demonstrated that ArfB can also act on ribosomes stalled on a 21-nucleotide rare-codon cluster, which extends beyond the A site^16^. The homologous ICT1 was also reported to act on ribosomes with long mRNAs^17^. It therefore remains unclear how ArfB recognizes stalled ribosomes with varying mRNA lengths and/or sequences.

In this work, we report biochemical and structural results revealing two modes of ArfB action. Biochemical data demonstrate that ArfB efficiently functions on mRNAs with a short overhang extending into the A site (+1 to +3 nucleotides beyond the P-site codon) and, surprisingly, on mRNAs that could fill the mRNA tunnel (+9 nucleotides beyond the P-site codon). The efficiency of ArfB is decreased on substantially longer mRNAs that are likely to extend beyond the mRNA tunnel (+21 nucleotides beyond the P site). We used cryogenic electron microscopy (cryo-EM) to visualize how ArfB acts on distinct 70S substrates. Extensive classification of two cryo-EM data sets resolves ensembles of 70S•ArfB structures with shorter and longer mRNAs. ArfB functions strictly as a monomer on a shorter +2 mRNA, whose overhang is stabilized by decoding center nucleotides A1492 and A1493. By contrast, dimeric ArfB stabilizes the longer +9 mRNA outside the mRNA tunnel. These findings allow to reconstruct two pathways for ArfB-mediated rescue of stalled ribosomes.

## Results and discussion

### ArfB functions on mRNAs extending into and beyond the A site

**Fig. 1:**
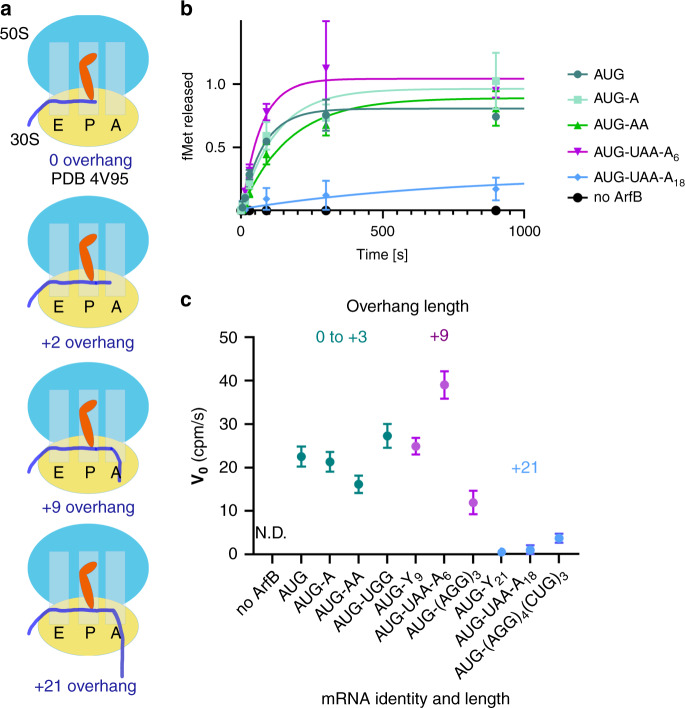
Catalytic activity of ArfB on 70S ribosomes with mRNAs of different lengths and sequences. **a** Schematic of ribosome complexes programmed with mRNAs that extend 0-21 nucleotides beyond the P site (AUG codon). **b** Time courses of ArfB-catalyzed [^35^S]-fMet release from 70S complexes with mRNAs with or without poly-adenosine overhangs. CPM readout was normalized relative to the signal for the mRNA with no overhang (AUG) at 1 h (*N* = 3 independent experiments). **c** Apparent rates (v_0_) of ArfB-catalyzed fMet release from ribosomes with mRNAs containing poly-pyrimidine (denoted Y), poly-A, and poly-arginine/leucine-encoding sequences (codons listed) of different lengths (N.D. not detected). (Error bars represent errors of fit from *N* = 3 independent experiments).

To measure ArfB function, we performed [^35^S]-N-formyl-methionine release experiments (see “Methods” and ref. ^18^) on *E. coli* 70S ribosomes programmed with mRNAs of varying lengths and sequences. We used three groups of mRNAs extending beyond the P-site AUG codon: (1) containing a short overhang of three or fewer nucleotides extending into the A site, (2) filling the mRNA tunnel with an overhang of +9 nucleotides, or (3) extending beyond the ~12-nucleotide mRNA tunnel with an overhang of +21 nucleotides^19–21^. The longer mRNAs contained a poly-arginine/leucine stalling site (AGG/CUG^16^), poly-lysine-encoding sequence (AAA codons), or a poly-pyrimidine sequence (Fig. 1a, Supplementary Table 1). Surprisingly, ArfB efficiently catalyzed release from ribosomes programmed with short and +9 mRNAs, whose sequences modestly affected release rates (Fig. 1c). The low nanomolar K_M_ for both short and +9 mRNAs (20 nM or lower; Supplementary Fig. 1) suggest efficient binding of ArfB to these substrates, likely commensurate with cellular concentrations of ArfB homologs^12,13^. ArfB had a reduced but detectable activity on a +21 mRNA with a poly-arginine stalling sequence (AGG)_4_ previously reported to be a substrate for ArfB (Fig. 1b, c^16^).

### Cryo-EM reveals three conformations of ArfB on the + 2 mRNA

**Fig. 2:**
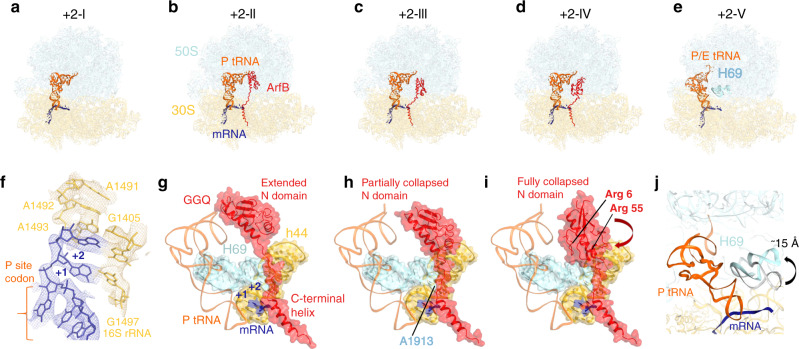
Cryo-EM structures of the 70S complex formed with +2 mRNA and ArfB. **a**–**e** Overall views of five cryo-EM structures with and without ArfB. **f** Cryo-EM density (mesh) showing the mRNA +2 overhang stabilized by decoding center nucleotides in Structures +2-I through +2-V (Structure + 2-II is shown; map was sharpened by a B-factor of −50 Å^2^). **g**–**i** Close-up view of structures +2-II, +2-III, and +2-IV with ArfB in the fully extended (**g**), partially collapsed (**h**) and fully collapsed (**i**) conformations, respectively. The arrow in panel i indicates rotation of N domain relative to that in panel **h**. **j** Rearrangement of 23S rRNA helix 69 (H69) in the rotated 70S ribosome without ArfB (+2-V) relative to H69 in Structure +2-IV (gray).

**Fig. 3:**
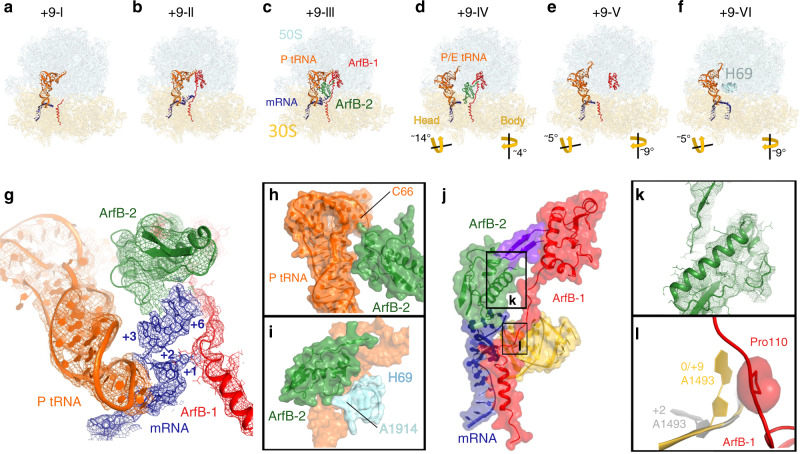
Cryo-EM structures of the 70S complex formed with +9 mRNA and ArfB. **a**–**f** Overall views of six cryo-EM structures with and without ArfB. **g** Cryo-EM density (mesh) showing mRNA (blue) outside the mRNA and interactions with P-site tRNA (orange), ArfB-1 (red), and ArfB-2 (green) (B = −25 Å^2^ except for ArfB-2 which was softened by B = 100 Å^2^). **h**–**j** Close-up views showing ArfB-2 interactions with P-tRNA, helix 69, ArfB-1 and mRNA. Two-strand β-sheet formed by ArfB-1 and ArfB-2 N-termini is in purple (**j**). **k** Cryo-EM density for ArfB-2 (B = 50 Å^2^). **l** Different conformations of the decoding center nucleotide 1493 in 70S•ArfB complexes formed on the +2 mRNA (gray) and +9 mRNA (yellow). Structure +9-III is shown in panels **g**–**l**.

To understand the structural basis for ArfB function on mRNAs of different lengths, we determined cryo-EM structures of 70S ribosomal complexes programmed with mRNAs containing a +2 (Fig. 2) or +9 (Fig. 3) poly-adenosine overhang. Maximum-likelihood classification revealed several structures in each dataset, featuring ribosomes in different conformations. Previous studies found that the ribosome adopts non-rotated and rotated conformations during canonical RF-catalyzed peptide release^22,23^. The non-rotated (classical) conformation is the primary substrate for RF binding^24^. It contains P-site tRNA, whose anticodon stem loop is bound to the mRNA codon on the small 30S subunit and the CCA end is in the P site of the large 50S subunit^25,26^. An ~10° rotation of the 30S subunit relative to the large subunit shifts the tRNA’s CCA end into the E site of the 50S subunit, so the tRNA adopts a hybrid P/E state. Intersubunit rotation prepares the ribosome for release-factor dissociation^27,28^ and recycling^29–31^.

The +2 dataset yielded five cryo-EM maps that suggest how ArfB functions on a short-mRNA substrate (Fig. 2, Supplementary Fig. 2, Supplementary Table 2). A 3.8-Å structure (+2-I) reports a non-rotated ribosome with the P-site tRNA and a vacant A site (Fig. 2a), resembling a pre-release or post-release state without a release factor^28^. Two 3.7 Å maps (+2-II and +2-IV) and a 3.8 Å map (+2-III) report non-rotated ribosomes with ArfB in different conformations (Fig. 2b–d). A 3.5-Å rotated 70S structure (+2-V) does not contain ArfB (Fig. 2e).

Structures +2-II through +2-IV bring insights into the dynamics of ArfB in the ribosomal A site (Figs. 2b–d and 2g–i). In Structure +2-II, the catalytic domain is in the PTC and C-terminal α-helix is in the mRNA tunnel, resembling ArfB in a complex with no mRNA overhang^15^. The catalytic^26^ GGQ^28^ motif is placed next to the terminal A76 of the P-site tRNA, consistent with catalytically engaged ArfB (Fig. 2g). The linker connecting the catalytic domain with the C-terminal helix (aa 101-112) contacts the tip of helix 69 (H69) of 23S rRNA at nucleotides A1913 and C1914 (Fig. 2g, h). In Structures +2-III and +2-IV, however, the catalytic domain is retracted from the PTC (Fig. 2h, i) and the GGQ loop is not resolved. The N-terminal domains are either partially (+2-III) or fully (+2-IV) collapsed onto H69, while the C-terminal α-helix remains in the mRNA entry tunnel. In the fully collapsed conformation, the N domain is shifted ~12 Å toward the 30S subunit A-site and rotated (Fig. 2i), placing a patch of positively charged residues (Arg6, His7, and Arg55) next to the phosphate backbone of H69 at C1914 and U1915 (Fig. 2h, i).

### Ribosomal decoding center stabilizes +2 mRNA

The +2 mRNA structures reveal that decoding center nucleotides rearrange to resolve the anticipated steric clash between the mRNA overhang and ArfB^15^. Here, A1492 and A1493 of 16S rRNA are within helix 44 and stack with the +2 nucleotide of the mRNA (Fig. 2f). The interaction shifts the overhang and widens the entry to the mRNA tunnel. This conformation of DC contrasts that in the crystal structure with no mRNA overhang, where A1493 is rotated by ~90° to bulge out of helix 44 and pack on Pro110 of the ArfB linker (Fig. 3l^15^). The differences reveal the plasticity of the decoding center, allowing ArfB to rescue stalled ribosomes that carry different mRNA overhangs.

The catalytically engaged Structure +2-II and previously unseen disengaged conformations of ArfB (+2-III and +2-IV) are consistent with a proposed mechanism for ArfB activation on stalled ribosomes^15^. The C-terminal helix acts as a sensor that binds a ribosome with a vacant mRNA tunnel or with a rearranged decoding center that accommodates a short mRNA overhang. Once the C terminus is anchored, the flexible ArfB linker allows the catalytic N domain to sample intersubunit space and insert into the PTC, releasing the peptide. This mechanism ensures recognition of the stalled ribosome before the catalytic domain is poised to act, preventing ArfB from spontaneously releasing peptides in actively translating ribosomes.

### Dimeric ArfB binds long +9 mRNA outside the mRNA tunnel

Cryo-EM analysis of the 70S complex formed with +9 mRNA yielded six ~3.5 Å structures (Supplementary Fig. 3; Supplementary Table 3), which reveal unexpected differences from the +2 complex. The mRNA tunnel of non-rotated 70S is occupied by the ArfB C-terminal helix, while the N domain is not resolved (+9-I; Fig. 3a), present as a monomer (+9-II; Fig. 3b) or present as a dimer (+9-III; Fig. 3c). The mRNA overhang is outside the mRNA tunnel and is stabilized by ArfB found predominantly in the dimeric state (+9-III; Fig. 3c). Unlike the +2 complex, rotated ribosome conformations are sampled either with ArfB (+9-IV and +9-V; Fig. 3d, e) or without ArfB (+9-VI; Fig. 3f).

Ribosome particle distribution reveals that non-rotated ribosomes with dimeric ArfB are two times more abundant than those with monomeric ArfB (Supplementary Figs. 4–5; Supplementary Tables 2-4). In Structure +9-III, the catalytic monomer (ArfB-1) is nearly identical to that formed on the short mRNAs or in Structure +9-II with monomeric ArfB (Figs. 2b, c; 3b, c). The second ArfB molecule in the intersubunit space (ArfB-2) is sandwiched between ArfB-1, P-site tRNA, H69 and mRNA (Fig. 3g–k). Cryo-EM density allows unambiguous assignment of secondary structure for ArfB-2 in Structure +9-III (Fig. 3k). Data classification revealed 8 additional classes with less-resolved ArfB-2 density, indicating that dynamic ArfB-2 samples different contacts with its interaction partners (Supplementary Fig. 6). In Structure +9-III, N-terminal residues of ArfB monomers interact forming a two-strand β-sheet (Fig. 3j; Supplementary Fig. 3e). ArfB-2 is further stabilized by interactions of a short helix (at Glu50 and Tyr51) with the minor groove of the acceptor arm of P-site tRNA (at C66 and C67) (Fig. 3h). The long helix of the N-terminal domain is docked between the tip of H69 (Fig. 3i) and the linker of ArfB-1 (at Lys103; Fig. 3j). The GGQ loop and the C-terminal tail are not resolved, consistent with structural flexibility of these regions in catalytically disengaged ArfB-2. ArfB-2 is flipped nearly 180 degrees relative to ArfB-1, so its C terminal region faces the 50S subunit, making ArfB-2 incompatible with binding the mRNA tunnel (Supplementary Fig. 3f).

ArfB-2 exposes a patch of positively-charged and hydrophobic residues (His62-Leu63, Lys73 and Arg90) to stabilize the bases of the mRNA overhang (positions +3 to +6). The mRNA is retracted from its canonical position in the mRNA tunnel by more than 20 Å (Fig. 3g, j; (measured at residue +4^32^)). To accommodate at the tightly packed A site, the mRNA takes a sharp turn immediately after the P-site codon, placing +1 and +2 nucleotides next to the anticodon stem loop of the P-site tRNA (Fig. 3g). The backbone of the mRNA overhang (+4 to +6) is placed next to the hydrophilic residues of ArfB-1 linker at Arg109, Thr111, and Arg112 (Fig. 3g and Supplementary 7a), suggesting that the catalytic monomer also contributes to mRNA stabilization outside the tunnel. Indeed, mRNA adopts a similar conformation in the less abundant Structure +9-II with monomeric ArfB (Fig. 3b). Conformation of the decoding center in the +9 structures differs from that formed on the +2 mRNA and resembles that in the crystal structure with no mRNA overhang^15^ (Figs. 2f; 3l).

Comparison of the +2 and +9 data sets reveals that ArfB dimerization is specific for the ribosomes with longer mRNA. First, extensive classification of the +2 complex data set, using the same algorithms as for the +9 data set, yielded exclusively monomeric and ArfB-vacant ribosomes, demonstrating that dimeric ArfB is beyond the detection limit in the 70S complex with a short mRNA overhang (Supplementary Fig. 4). Second, non-rotated ribosomes with ArfB dimer are substantially more abundant than vacant or monomeric-ArfB-bound 70S (Supplementary Fig. 4, Supplementary Fig. 5), suggesting that longer mRNA increases the affinity of ArfB dimer to the ribosome. Indeed, NMR studies report monomeric ArfB in solution, indicating that the requirements for dimerization are not met for isolated ArfB^10^. Position of mRNA outside the tunnel suggests that ArfB binding correlates with large-scale dynamics of mRNA. The mRNA tunnel is formed between the head and body of the 30S subunit, which rearrange during translation elongation^33^. Rotation of the head relative to the body (also known as “head swivel”) can achieve ~20°. The opening between these 30S domains allows mRNA to sample conformations outside the mRNA tunnel^34,35^. ArfB dimerization likely shifts the mRNA dynamic equilibrium by fixing longer mRNAs outside the mRNA tunnel. This stabilization would allow ArfB-1 to achieve a catalytically active conformation and release the nascent peptide from P-site tRNA.

### ArfB and 70S intersubunit rotation

+2 and +9 complexes differ in the occupancies of ArfB on the rotated 70S ribosome, suggesting that ArfB dissociation during intersubunit rotation occurs differently. Similarly to the post-termination 70S complexes with RF2^28^, the A site of the rotated Structure +2-V (~7°) is vacant (Fig. 2e and Supplementary Fig. 2). The head of the small subunit is modestly swiveled (~5°). The central intersubunit bridge is disassembled, as H69 is disengaged from its canonical position in the 30S decoding center and is packed onto the 50S subunit (Fig. 2j). In this position, H69 would clash with ArfB (Supplementary Fig. 3d), consistent with dissociation of ArfB upon intersubunit rotation.

By contrast, ribosomes programmed with the longer +9 mRNA sample partially (4°) and fully (9°) rotated 70S conformations, which feature ArfB in the A site (Fig. 3d, e) or a vacant A site (Fig. 3f). Classification revealed ten maps (Supplementary Fig. 3 and Supplementary Table 3), three of which were modeled as representative states (Fig. 3d–f). The partially rotated conformations (Fig. 3d) have the 30S head swiveled by ~14° toward the 50S subunit, resembling mid-rotated ribosome in EF-G-bound translocation complexes^36^. In both partially and fully rotated ribosomes with ArfB, the positions and occupancies of the catalytic ArfB and ArfB-2 vary (Supplementary Table 3, Supplementary Figs. 5, 6). In nine maps with ArfB, the C-terminal helix is less resolved in the mRNA tunnel than in the non-rotated ribosome (e.g., Structure +9-IV; Fig. 3d, e; Supplementary Fig. 7), suggesting destabilization of the helix upon intersubunit rotation and head swivel. The mRNA overhang is unresolved in all but one map, in which mRNA is bound to dimeric ArfB (Supplementary Table 3). Rotated 70S without ArfB contains mRNA in the tunnel (Structure + 9-VI; Fig. 3f and Supplementary 7c), consistent with mRNA re-entering the tunnel upon ArfB dissociation. Similar to the +2 mRNA, the ArfB-vacant rotated 70S ribosome features H69 detached from the decoding center and pointing toward the A site. In this position, H69 would sterically clash with ArfB (Fig. 3f and Supplementary Fig. 3d).

Ribosome particle distribution in the +9 data set reveals that the occupancy of ArfB dimer decreases upon intersubunit rotation (Supplementary Table 4; Supplementary Fig. 5). The occupancy of monomeric ArfB, however is higher on partially and fully rotated ribosomes (Supplementary Fig. 4), suggesting that the ArfB dimer may dissociate step-wise via monomeric states. Dissociation of the catalytic ArfB is likely inefficient because it is stabilized by the displaced mRNA and/or by A1493 (Fig. 3l). Nevertheless, the appearance of rotated ribosomes without ArfB (+9-IV; Fig. 3f and Supplementary Fig. 3) is consistent with ArfB dissociation upon intersubunit rotation. Thus, in both +2 and +9 datasets, intersubunit rotation is correlated with ArfB dissociation, similarly to canonical termination complexes with RF1 and RF2^27,28^. Stalled ribosomes with longer mRNAs appear to represent a challenging substrate for dimeric ArfB dissociation after peptide release. GTPase release factor RF3, which stabilizes the rotated conformation^27,29,30^, could assist ArfB dissociation in the cell, echoing the role of RF3 in RF1 dissociation from post-termination ribosomes^22,27^.

### ArfB mechanism: two modes of ribosome rescue

**Fig. 4:**
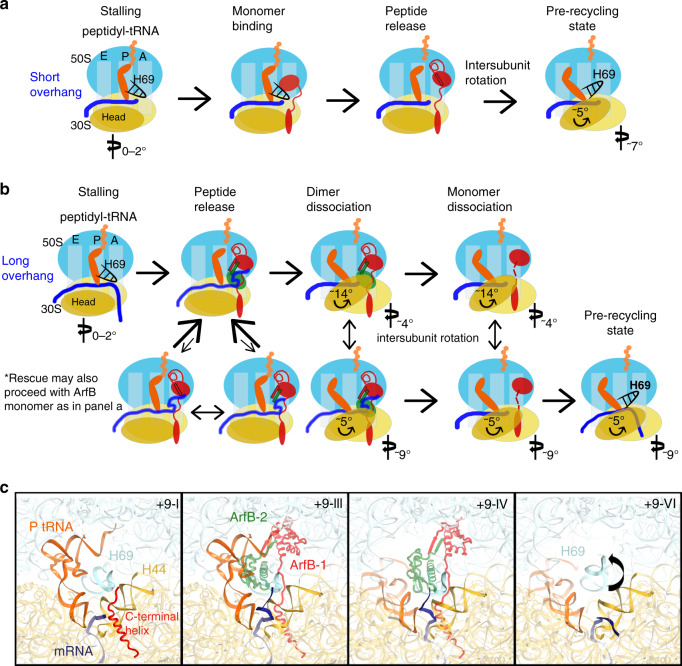
Proposed mechanism of ArfB-mediated rescue of ribosomes stalled on short and longer mRNAs. **a** Schematic of ArfB-mediated rescue of ribosomes stalled on mRNAs extending by a few nucleotides beyond the P site. **b** Schematic of ArfB-mediated rescue of ribosomes stalled on longer mRNAs extending into the mRNA tunnel. **c** Close-up views of cryo-EM structures +9-I, +9-III, +9-IV, and +9-VI showing rearrangements of P-tRNA, ArfB, mRNA, and H69.

Our findings reveal that ArfB has two modes of function depending on the length of mRNA in a stalled ribosome, and suggest the structural mechanisms for ArfB-mediated ribosome rescue (Fig. 4). Upon stalling on a truncated mRNA or a rare-codon cluster, the ribosome adopts a non-rotated conformation with peptidyl-tRNA in the P site. Truncation of mRNA in the A site (as in the 0 or +2 complexes) or a rare codon prevent efficient mRNA decoding by aminoacyl-tRNA, leaving the vacant A site accessible to ArfB. Short mRNA overhangs can be stabilized by the decoding center nucleotides, permitting entry of the C-terminal tail of ArfB into the mRNA tunnel (Fig. 4a). Anchoring the C-terminal helix in the tunnel exposes the N-terminal domain, connected via a flexible linker, to explore the intersubunit space in the vicinity of the A site. Mutational studies demonstrate that the linker length and C-terminal residues are critical for ArfB binding and catalysis^10,16^, suggesting that C-terminal binding precedes the insertion of the catalytic domain into the PTC. Our structures +2-III and +2-IV raise a possibility that transient interactions of the N-terminal domain with H69 outside the PTC might precede or coincide with C-terminus binding in the tunnel. However, the early steps and mechanism by which the flexible C-terminus recognizes the mRNA tunnel remain to be elucidated. Subsequent insertion of the N-terminal domain into the peptidyl transferase center positions the universally conserved catalytic GGQ motif next to the scissile ester bond of peptidyl-tRNA, similarly to those in RF-bound structures (reviewed in refs. ^1–5^). Deacylation of P-site tRNA allows the ribosome to spontaneously sample intersubunit rotation^37^, bringing the deacylated tRNA into the E site of the large subunit^38^. Our structures demonstrate that ArfB occupancy decreases upon intersubunit rotation (Supplementary Fig. 4 and Supplementary Fig. 5), consistent with ArfB dissociation. Structures +2-II through +2-IV are consistent with step-wise dissociation of ArfB from the ribosome, with the N-terminal domain dissociating before the C-terminal tail. Movement of H69 and rearrangement of ribosomal subunits, including head rotation, likely destabilize ArfB leading to its dissociation (Supplementary Fig. 3d). The rotated 70S is a substrate for recycling factor RRF^39^, which splits the ribosomes into the subunits, preparing them for translation of other mRNAs.

The evolution of structural states on ribosomes with longer-mRNA substrates is different, as expected from steric incompatibility of mRNA and ArfB in the mRNA tunnel (Fig. 4b, c). Here, downstream mRNA is stabilized outside the tunnel by an additional copy of ArfB, allowing the catalytic ArfB to perform its function. Thus, ArfB is an unusual enzyme that efficiently recognizes two groups of substrates via distinct multimerization states. Observation of mRNA dynamics and positions outside the mRNA tunnel, however, is not unprecedented. The majority of cryo-EM and crystal structures of ribosomes report a poorly ordered A-site codon in the absence of A-site tRNA or protein factors, and do not resolve mRNA in the entry tunnel. This indicates that mRNA downstream the P-site codon is conformationally unstable in the absence of a stabilizing A-site interaction. Furthermore, downstream mRNA with high propensity for secondary-structure formation can depart from the mRNA tunnel and fold in the A site^34,40^. It is possible therefore, that in addition to the truncated and rare-codon substrates, ArfB also recognizes no-go complexes with structured mRNA downstream the tunnel. By contrast, the catalytic activity of ArfB is low if mRNA is efficiently decoded by aminoacyl-tRNAs and is stabilized in the mRNA tunnel (Fig. 1b, c), preventing codon-independent peptide release on actively translating ribosomes. Dimerization-dependent rescue may underlie an expanded repertoire of ArfB targets upon upregulation of ArfB under stress or other conditions. Nevertheless, our findings do not rule out that ArfB could function as a monomer on some stalled ribosomes in a mRNA-sequence-dependent manner (Fig. 4b), as suggested by the study published while our manuscript was under review^41^.

Our findings shed light on a broader range of rescue mechanisms, reconciling the conflicting structural and biochemical data for other rescue pathways (Supplementary Fig. 8). In bacteria, ArfB (Supplementary Fig. 8a) is one of the three well-known pathways of stalled-ribosome rescue, which also include alternative rescue factor A with RF2 (ArfA•RF2; Supplementary Fig. 8b) and trans-translation by SmpB•tmRNA (Supplementary Fig. 8c)^7,9^. In eukaryotes, the Dom34/Pelota•Hbs1 complex (Supplementary Fig. 8d), which structurally resembles the release factor complex eRF1•eRF3, is the predominant rescue mechanism for cytoplasmic ribosomes^42–44^, whereas ArfB-like ICT1 rescues mitochondrial ribosomes^12,13^. Previous structural studies found that these pathways involve proteins that recognize the vacant mRNA tunnel. ArfA binds the mRNA tunnel to assist RF2 in releasing the nascent peptide^45–49^. In trans-translation, small protein B inserts into the tunnel and assists tmRNA to continue translation of a stalled protein targeted for degradation^50^. Dom34 inserts a β-hairpin loop into the mRNA tunnel to release peptidyl-tRNA from the ribosome^51,52^. Biochemical studies, however, have demonstrated that these pathways can also function on longer mRNAs. ArfA and SmpB function on +6 mRNAs, but their efficiencies are reduced on +9 substrates^53,54^. Dom34 acts to rescue ribosomes stalled on poly-A sequences^43,55^, echoing the roles of ArfB on rare-codon clusters. These data suggest that trans-translation and ArfA-mediated release in bacteria, as well as Dom34-mediated ribosome rescue in eukaryotes can occur when mRNA spontaneously exits the mRNA tunnel. While ArfB forms an mRNA-stabilizing homodimer, the other rescue pathways are not known to involve dimer formation. Isolated mitochondrial homolog ICT1 was reported to dimerize in solution^56^, but it remains to be shown whether ICT1 dimerizes to rescue stalled mitochondrial ribosomes. Cryo-EM structure of Dom34 bound the 80S ribosome with a poly-A-containing mRNA did not resolve mRNA beyond the P-site codon^52^, suggesting that Dom34 can efficiently outcompete mRNA that can stochastically leave the mRNA tunnel. Structural studies of trans-translation, ArfA, and ICT1 on longer mRNAs will help clarify how these rescue pathways function on mRNAs of different lengths and sequences.

## Methods

### Preparation of 70S complexes with ArfB

Full-length *E. coli* ArfB (YaeJ) without tags was subcloned into a pET24-b(+) plasmid from the ArfB-encoding ASKA construct obtained from the National Bioresource Project (Japan). The protein was overexpressed in *E. coli* BLR (DE3) (Novagen) cells. Upon achieving an optical density of 0.8, the cells were induced with 1 mM IPTG and incubated at 16 °C overnight in an orbital shaker. Cells were pelleted by spinning at 5000 rpm for 20 min and resuspended with buffer A (50 mM Tris pH 8.0, 50 mM ammonium sulfate, 100 mM potassium chloride, and 5 mM 2-mercaptoethanol). Cells were lysed, and the lysate was clarified by spinning at 18,000 rpm for 20 min. The supernatant was loaded onto a Mono-S 5/50 GL column pre-equilibrated with buffer A and eluted with buffer B (50 mM Tris pH 8.0, 50 mM ammonium sulfate, 1 M potassium chloride, and 5 mM 2-mercaptoethanol). Fractions containing ArfB were pooled and re-applied to the Mono-S column. Fractions containing ArfB were pooled and exchanged into buffer C (50 mM Tris pH 8.0, 300 mM potassium chloride, 10% glycerol, and 5 mM 2-mercaptoethanol). The sample was applied to a HiLoad 16/60 size exclusion column (prep grade) pre-equilibrated in buffer C. Collected fractions containing pure ArfB (>95% estimated by PAGE electrophoresis) were concentrated and exchanged into storage buffer (20 mM Tris acetate pH 7.0, 10 mM ammonium acetate, 50 mM potassium acetate, and 10 mM magnesium acetate) using centrifugal filters. *E. coli* tRNA^fMet^ (Chemical Block) was aminoacylated as described^18^. 70S ribosomes were prepared from *E. coli* (MRE600) as described^18^. Ribosomes were stored in the ribosome storage buffer (20 mM Tris hydrochloride pH 7.0, 100 mM ammonium chloride, 12.5 mM magnesium chloride, 0.5 mM ethylenediaminetetraacetic acid, and 6 mM 2-mercaptoethanol) at −80 °C. Model mRNAs for this study (Supplementary Table 1), containing the Shine-Dalgarno sequence and a spacer to position the AUG codon in the P site (GGCAAGGAGGUAAAAAUG-overhang), were synthesized by IDT.

Biochemical activity of ArfB was measured using an in vitro assay that records release of [^35^S]-formyl-methionine from the 70S ribosome bound with [^35^S]-fMet-tRNA^fMet^ in the P site, as described^18^. Pre-termination complex was prepared using 100 nM *E. coli* 70S ribosomes (all concentrations are for the final reaction mixture upon initiation of the release reaction), 4.95 µM mRNA, and 275 nM [^35^S]-fMet-tRNA^fMet^ ([^35^S]-methionine from Perkin Elmer). Concentrations were determined using Nanodrop 2000c Spectrophotometer. The reaction was carried out on ice with 10 µM ArfB, with 5-uL aliquots taken at 0, 6, 15, 30, 90, 300, 900, 1800, and 3600 s and quenched in 0.1 M HCl to represent reaction time points. Formyl-methionine was extracted by ethyl acetate and radioactivity was measured in a scintillation counter (Beckman Coulter, Inc) using a scintillation cocktail (Econo-safe). All time-progress curve measurements were performed 3 times and k_obs_ were obtained by fitting single-exponential curves using GraphPad Prism8: *y* = *y*_0_ + (Plateau-*y*_0_)*(1−e^−kx^).

For cryo-EM studies, two 70S•tRNA^fMet^•mRNA•ArfB complexes were assembled as follows. 400 nM 70S ribosome (all concentrations are specified for the final complex solution) was combined with 12 µM mRNA (mRNA + 2: GGCAAGGAGGUAAAAAUG-AA; mRNA+9: GGCAAGGAGGUAAAAAUG-UAAAAAAAA) in buffer containing 20 mM Tris-acetate pH 7.0, 100 mM ammonium acetate, and 20 mM magnesium acetate. After vortexing and incubation at 37 °C for 2 min, 600 nM tRNA^fMet^ was added, vortexed, and incubated at 37 °C for 5 min. Four micromolar ArfB was added to the 70S complex, vortexed, and incubated at 37 °C for 15 min. The sample (70S•tRNA^fMet^•mRNA•ArfB complex) was kept on ice during grid preparation and plunging.

### Cryo-EM and image processing

Holey-carbon grids (QUANTIFOIL R 2/1, Cu 200, 2 nm C grid, Quantifoil Micro Tools) coated with carbon were glow discharged at 20 mA with negative polarity for 60 s in a PELCO easiGlow glow discharge unit. 2.8 µl of the 70S•mRNA•tRNA^fMet^•ArfB complex was applied to the grids. Grids were blotted at blotting force 8 for 6 s at 20 °C, 100% humidity and plunged into liquid ethane using a Vitrobot MK4 (FEI). Grids were stored in liquid nitrogen. Grid preparation for both data sets was identical.

+9 mRNA: A dataset of 197,913 particles was collected on a Titan Krios (FEI) microscope (operating at 300 kV) equipped with a K2 Summit camera system (Gatan), with 0.6 to 1.8 µm defocus. Multi-shot data collection was performed by collecting 4 exposures from 4 holes using SerialEM^57^ with beam-image shift. Built-in functions of SerialEM were used in performing coma-free alignment. The “Coma vs. Image Shift” function was used to compensate for dynamic beam-tilt occurring during each exposure. Multi-hole/multi-shot configuration was selected from “Multiple Record Setup Dialog” to dynamically adjust the beam tilt and mechanical drift was minimized by applying backlash-corrected compensation for each stage adjustment. 1,777 movies were collected. Each exposure (35 frames per movie) was acquired with continuous frame streaming, yielding a total dose of 49.6 e/Å. The nominal magnification was 130,000 and the calibrated super-resolution pixel size was 0.521 Å. Movies with ice contamination or image recording defects were discarded, yielding 1521 movies for data processing. IMOD^58^ was used to align movies. Resulting images were binned to pixel size 1.042 Å (termed unbinned or 1× binned). cisTEM^59^ was used to determine defocus values for each resulting frame average, for particle picking, for 2D classification to remove poorly resolved and 50S particles, and for generating an ab initio model as a template during alignment. The stack and particle parameter files were assembled in cisTEM with the binnings of 1×, 2×, and 4× (box size of 360 for unbinned stack).

+2 mRNA: A dataset of 90,503 particles was collected in a similar manner to the +9 mRNA dataset on the Talos-Arctica (FEI) microscope (operating 200 kV) equipped with a K3 camera system (Gatan), with 0.5 to 1.5 µm defocus. Multishot data collection was performed by collecting 4 exposures for 4 holes at a time, following the same setup as described above. 1504 movies were collected. Each exposure (27 frames per movie) was acquired with continuous frame streaming, yielding a total dose of 30.5 e/ Å^2^. The nominal magnification was 45,000 and the calibrated super-resolution pixel size was 0.435 Å. 1460 movies remained after discarding those with obvious defects due from ice contamination. The frames for each movie were processed as described above with the unbinned or 1× pixel size measuring 0.87 Å. cisTEM was used to determine defocus values, pick particles, 2D classify, and for generating an ab initio model to use for a template during alignment. The stack and particle parameter files were assembled in cisTEM with the binnings of 1×, 2×, and 4× (box size of 440 for unbinned stack).

+9 mRNA: FrealignX was used for particle alignment, refinement, and final reconstruction steps, and Frealign v9.11 was used for classification steps^60,61^. The 4×-binned image stack (197,913 particles) was initially aligned to an ab initio reference model—generated from 50% of the particles from this data set—using 3 cycles of mode 3 (global search) alignment, including data from 25 Å to 300 Å resolution range. Using the 4×-binned stack, the particles were refined using mode 1 by gradually (1-5 Å per step) increasing the high-resolution limit from 25 to 9 Å (3 cycles for each resolution limit). 3D density reconstruction was obtained using 60% of particles with highest scores. The map contained density for the P-tRNA, mRNA, and ArfB. Conversion of parameter file from FrealignX to Frealign for classification was performed by removing column twelve, which contains phase shift information (not applicable as no phase plate was used) and adding an absolute magnification value. Reverse conversion from Frealign to FrealignX for refinement was performed automatically by FrealignX.

Data classification was performed as illustrated in Supplementary Fig. 3a, using a spherical focused mask with a 40 Å radius, centered in the intersubunit space and covering the A site and part of the P site (Supplementary Fig. 2d). The particle stack was classified into 4 classes using the high-resolution limit of 12 Å. The classification resulted in two classes of nonrotated ribosomes with ArfB bound (Classes 1 and 2) and two classes of rotated ribosomes with ArfB (Classes 3 and 4). All classes were reconstructed using the unbinned stack. Classes 3 and 4 were subclassified using the same 40 Å focused mask into 6 and 4 classes, respectively. This subclassification revealed several ArfB-containing states (Supplementary Table 3), which were subjected to final refinement with the high-resolution limit of 6 Å (Fig. 4b). Three maps were fitted with structural models to yield Structures +9-IV (3.7 Å), + 9-V (3.3 Å), and +9-VI (3.2 Å). Subclassification of Class 1 into 6 classes using the 40 Å focused mask produced multiple states, which were refined and fit as described for Classes 3 and 4 (Supplementary Table 5). Two maps containing ArfB yielded Structures +9-I (3.2 Å) and +9-II (3.5 Å). Class 2 was subclassified using a 30 Å focused mask that covered the 50S A and P sites, as well as the PTC (Supplementary Fig. 5d). This classification yielded Structure +9-III (3.3 Å) after final refinement with a high-resolution limit of 6 Å.

+2 mRNA: Data processing of the +2 mRNA data set followed a similar workflow as the +9 mRNA data set. In short, classification on the full stack using the same 40 Å spherical focused mask for 100 cycles revealed two classes: monomer ArfB bound to classical ribosomes and no ArfB bound to hybrid state ribosomes. The ArfB containing class was subclassified again with the same mask which produced three classes: no ArfB classical ribosomes, monomer ArfB bound classical ribosomes (3.7 Å + 2-II, high resolution limit of 6 Å), and collapsed, monomer ArfB bound classical ribosomes. The collapsed, monomer class was further classified using a 35 Å spherical focused mask around the peptidyl transferase center and refined to produce 3.8 Å structure +2-III containing partially collapsed ArfB and 3.7 Å structure +2-IV containing fully collapsed ArfB (high resolution limit of 6 Å). The overall workflow for classification of the +2 mRNA data set can be seen in Supplementary Fig. 2a, and the discussed classes are described in Supplementary Table 2.

The resulting reconstructions varied from 3.3 Å (+9-III) to 3.8 Å (+2-III) (Fourier Shell Correlation (FSC) = 0.143). Cryo-EM maps were B-factor sharpened in bfactor.exe (part of Frealign distribution) using different B-factor values (Supplementary Table 5) and then used for model building and structural refinements. FSC curves were calculated by FrealignX for even and odd particle half-sets (Supplementary Fig. 2b and Supplementary Fig. 3b).

### Model building and refinement

Cryo-EM Structure I of 70S•fMet-tRNA^fMet^•RF2^28^ and the crystal structure of 70S•tRNA^fMet^•ArfB^15^ were used to create initial models for structural refinements of the non-rotated ribosomes. Cryo-EM Structure V of 70S•fMet-tRNA^fMet^^28^ was used to create initial models of the rotated ribosomes. Chimera^62^ was used for fitting the 50S subunit, 30S head with mRNA, 30S body, tRNA, and ArfB. N-terminal domain of ArfB-2 was fitted into density as a rigid body, excluding the GGQ loop and the C-terminal tail. Local model fitting was performed in PyMOL for areas that differed from the starting model, such as A-site mRNA and N-terminal residues of ArfB-2^63^.

The structural models were refined by real-space simulated-annealing refinement using atomic electron scattering factors in RSRef and Phenix, as described^32,64–66^. During refinement in RSRef, secondary-structure restraints, comprising hydrogen bonding restraints for ribosomal proteins and ArfB, and base-pairing restraints for RNA molecules, were employed as described^67^. Refinement parameters, such as the relative weighting of stereochemical restraints and experimental energy term, were optimized to produce the models with good stereochemistry that closely agree with the corresponding maps. The structures were next refined using phenix.real_space_refine^68^, followed by a round of refinement in RSRef with harmonic restraints applied to all proteins to preserve the backbone geometry. In the final step, structure B-factors were refined in Phenix against the respective maps. The resulting structural models have good stereochemical parameters, evidenced by low deviation from ideal bond lengths and angles, high correlation coefficients, and low real space R factors (Supplementary Table 5). Structure quality was validated using MolProbity^69^.

Figures were prepared in Chimera, Graphpad Prism8, and PyMOL.

### Description of structures

Cryo-EM maps for all structures were deposited in EMDB. PDB structures were deposited in RCSB, except for Structures +2-I, +2-V, +9-I, +9-V, and +9-VI, which are similar to published structures, as described below.

Structure +2-I is a non-rotated 70S•tRNAfMet without ArfB. The +2 mRNA assumes an identical conformation across all five +2 structures, Structure +2-I can be recreated in PyMOL by removing ArfB from +2-II.

Structure +2-V is a rotated 70S•tRNAfMet with P/E tRNA and helix 69 (H69) disengaged from the decoding center, similar to the reported Structure V (without RF2) in PDB:6OGI^70^, except the different mRNA identity and conformation of the decoding center (see description for +2-I).

Structure +9-I is a non-rotated 70S•tRNAfMet with the C-terminus of ArfB (residues 107–133) resolved in the mRNA tunnel. No density for the N domain of ArfB or mRNA residues beyond the P site were observed. +9-I is therefore similar to +9-III without mRNA after the P site, ArfB-2, and ArfB-1 residues 1-106.

Structure +9-V is a rotated 70S•tRNAfMet with P/E tRNA, mRNA resolved through the P site, and ArfB N and C domains (residues 1-20, 36-100, and 111-133).

Structure +9-VI is similar to +2-V, except that mRNA resides inside the mRNA tunnel as in PDB PDB:4V6G (Supplementary Fig. 7c^71^).

## Supplementary information

Supplementary Information

## Data Availability

The data that support this work is available from the corresponding author upon reasonable request. The models and cryo-EM maps described in this study are available from RCSB PDB and EMDB with the following accession codes: +2-I (EMD-22465), +2-II (EMD-22459, PDB 7JSS), +2-III (EMD-22461, PDB 7JSW), +2-IV (EMD-22464, PDB 7JSZ), +2-V (EMD-22467). +9-I (EMD-22468), +9-II (EMD-22469, PDB 7JT2), +9-III (EMD-22466, PDB 7JT1), +9-IV (EMD-22472, PDB 7JT3), +9-V (EMD-22471), and +9-VI (EMD-22470). Source data are provided with this paper.

## Source data

Source Data
