# Supplementary material for: ArfB can displace mRNA to rescue stalled ribosomes
Source: Nat Commun. 2020 Nov 3;11:5552. doi: 10.1038/s41467-020-19370-z (PMC7641280; doi:10.1038/s41467-020-19370-z)
Supplement: Supplementary file 1 — Supplementary Information [file 41467_2020_19370_MOESM1_ESM.pdf]

Supplementary Information

Supplementary Table 1. Sequences of mRNAs used in this work

mRNA Name:	Overhang length	mRNA sequence
Poly-Lysine	0	GGC-AAG-GAG-GUA-AAA-AUG
	1	GGC-AAG-GAG-GUA-AAA-AUG-A
	2	GGC-AAG-GAG-GUA-AAA-AUG-AA
	9	GGC-AAG-GAG-GUA-AAA-AUG-UAA-(AAA)$_2$
	21	GGC-AAG-GAG-GUA-AAA-AUG-UAA-(AAA)$_6$
Poly-Arginine/Leucine	9	GGC-AAG-GAG-GUA-AAA-AUG-(AGG)$_3$
	21	GGC-AAG-GAG-GUA-AAA-AUG-(AGG)$_4$-(CUG)$_3$
Poly-Pyrimidine	9	GGC-AAG-GAG-GUA-AAA-AUG-AUC-ACC-AUC
	21	GGC-AAG-GAG-GUA-AAA-AUG-AUC-ACC-AUC-AAC-ACU-UCU-CAC
Tryptophan	3	GGC-AAG-GAG-GUA-AAA-AUG-UGG

Supplementary Table 2. Description of cryo-EM classes for the +2 mRNA data set. 30S body and head rotation was calculated relative to Structure +9-III

Class Description (Structure)	Degrees of Rotation	Number of Particles	Resolution (Å)
70S classical, Empty A site (+2-I)	Head: ~1° Body: ~2°	9,320	3.8
ArfB monomer extended (+2-II)		10,953	3.7
ArfB monomer partial collapse (+2-III)	Head: ~1° Body: ~3°	7,464	3.8
ArfB monomer full collapse (+2-IV)		12,528	3.7
70S rotated, Empty A site (+2-V)	Head: ~5° Body: ~7°	48,419	3.5

Supplementary Table 3. Description of cryo-EM classes for the +9 mRNA data set. 30S body and head rotation was calculated relative to Structure +9-III

Class Description (Structure)	30S Rotation	Class Number	Number of Particles	Resolution (Å)
ArfB C-terminal tail only (+9-I)	Head: ~0° Body: ~0°	1.1	9,841	3.4
ArfB monomer: displaced N domain		1.2	8,887	3.4
ArfB monomer (+9-II)		1.3	8,184	3.5
ArfB dimer		1.4	8,588	3.5
ArfB dimer sampling		1.5 & 1.6	14,908	3.2
ArfB dimer	Head: ~0° Body: ~0°	2.1	9,679	3.5
ArfB dimer (+9-III)		2.2	14,367	3.3
ArfB dimer		2.3	10,864	3.4
Empty A site	Head: ~14° Body: ~4°	3.1	4,567	3.9
ArfB monomer, linker unresolved		3.2	5,211	3.6
ArfB monomer, linker unresolved		3.3	6,461	3.6
ArfB dimer (+9-IV)		3.4	5,711	3.7
ArfB dimer		3.5	4,382	4.0
ArfB dimer		3.6	4,896	3.8
Empty A site (+9-VI)	Head: ~5° Body: ~9°	4.1	16,184	3.2
ArfB monomer, linker unresolved		4.2	12,626	3.4
ArfB monomer, linker unresolved (+9-V)		4.3	12,969	3.3
ArfB dimer with mRNA resolved		4.4	12,238	3.3

Supplementary Table 4. Cryo-EM particle distributions in the +9 mRNA data set. Monomer was defined as either the N or C domain density with no evidence of ArfB-2. Dimer was defined as monomer plus any component of ArfB-2 (see also Supplementary Fig. 5).

State	Particles (#)	Particles (%)
Non-rotated Ribosome		
Empty A site	0	0
ArfB monomer	26,912	13.6
ArfB dimer	58,406	29.6
Rotated Ribosome		
Empty A site	20,751	10.5
ArfB monomer	37,267	18.9
ArfB dimer	27,227	13.8
Unassigned Particles (<50% occupancy in either class)	27,350	13.8
Total: ArfB-bound ribosomes	139,971	70.8
Total Particles	197,613	100

Supplementary Table 5. Refinement statistics for cryo-EM structures of 70S complexes with ArfB and +2 or +9 mRNAs.

	+2-I	+2-II	+2-III	+2-IV	+2-V	+9-I	+9-II	+9-III	+9-IV	+9-V	+9-VI
Data collection and processing											
Magnification	45,000x	45,000x	45,000x	45,000x	45,000x	130,000 x	130,000 x	130,000 x	130,000 x	130,000 x	130,000 x
Voltage (kV)	200	200	200	200	200	300	300	300	300	300	300
Electron exposure (e–/Å^2)	30.5	30.5	30.5	30.5	30.5	49.6	49.6	49.6	49.6	49.6	49.6
Defocus range (μm)	0.5-1.5	0.5-1.5	0.5-1.5	0.5-1.5	0.5-1.5	0.6-1.8	0.6-1.8	0.6-1.8	0.6-1.8	0.6-1.8	0.6-1.8
Pixel size (Å)	0.87	0.87	0.87	0.87	0.87	1.042	1.042	1.042	1.042	1.042	1.042
Symmetry imposed	C1	C1	C1	C1	C1	C1	C1	C1	C1	C1	C1
Initial particle images (no.)	90,503	90,503	90,503	90,503	90,503	197,613	197,613	197,613	197,613	197,613	197,613
Final particle images (no.)					48,419	9,841	8,184	14,367	5,711	12,969	16,184
Map resolution (Å)**	3.7	3.7	3.8	3.7	3.7	3.4	3.7	3.3	3.7	3.3	3.2
FSC threshold	0.143	0.143	0.143	0.143	0.143	0.143	0.143	0.143	0.143	0.143	0.143
Map resolution range (Å)	3.5 - >6	3.0 - >5	3.5 - >6	3.5 - >5	3.0 - >5	3.0 - >5	3.0 - >6	2.8 - >6	3.5 - >6	3.0- >6	3.0 - >6
Refinement											
Initial model used (PDB code)	6OG7 4V95	6OG7 4V95	6OG7 4V95	6OG7	6OG7	6OG7	6OG7 4V95	6OG7 4V95	6OG7 4V95	6OG7 4V95	6OG7
Model resolution (Å)	3.8	3.7	3.8	3.7	3.5	3.4	3.5	3.3	3.7	3.3	3.2
Correlation Coefficient (cc_mask)*	0.84	0.85	0.83	0.84	0.87	0.83	0.82	0.76	0.78	0.80	0.85
Real space R-factor †	0.22	0.22	0.20	0.21	0.21	0.25	0.22	0.23	0.24	0.23	0.26
Map sharpening *B* factor (Å^2)	0	-50	0	0	0	+50	0	0	0	+25	+100
Model composition*											
Non-hydrogen atoms	143,859	144,890	144,80	147,862	143,859	147,069	145,985	145,600	148,597	147,703	146,907
Protein residues	5,617	5,749	5,737	6,144	5,617	6,050	5,880	5,828	6,249	6,123	6,023
RNA residues	4,655	4,655	4,655	4,655	4,655	4,653	4,660	4,659	4,652	4.656	4,655
B factors (Å^2)*											
Protein	220.63	258.42	205.23	227.59	239.39	208.07	165.04	95.50	217.81	249.40	267.00
RNA	232.75	277.84	223.63	219.40	243.33	203.49	169.25	103.03	211.13	224.79	259.56
R.m.s. deviations*,§											
Bond lengths (Å)	0.008	0.007	0.006	0.005	0.008	0.006	0.007	0.007	0.006	0.006	0.006
Bond angles (°)	1.0	0.9	0.09	1.0	1.0	0.9	0.9	1.0	0.9	0.9	0.9
Validation*											
MolProbity score	2.43	2.43	2.47	2.30	2.46	2.27	2.22	2.22	2.34	2.17	2.22
Clashscore	16.06	15.9	17.13	12.56	15.51	12.79	12.56	13.6	13.09	12.81	13.19
Poor rotamers (%)	1.33	0.60	0.60	0.88	1.44	0.87	0.98	1.15	1.20	0.50	0.67
Ramachandran plot*											
Favored (%)	86.99	81.04	79.93	83.27	85.87	85.50	87.73	90.71	85.87	89.96	88.48
Allowed (%)	11.15	16.73	16.35	13.38	12.27	14.13	10.78	9.29	11.90	91.08	10.78
Disallowed (%)	1.86	2.23	3.72	3.35	1.86	0.37	1.49	0.0	2.23	1.12	0.74
Validation (RNA)*											
Good sugar pucker (%)	99.5	99.8	99.8	99.8	99.5	99.9	99.7	99.7	99.8	99.8	99.7
Good backbone (%)#	84.1	85.3	82.0	85.0	84.1	85.4	84.2	84.3	84.4	84.8	84.3

** from Frealign (FSC_part)
* from Phenix
† from RSRef
§ root mean square deviations
# RNA backbone suites that fall into recognized rotamer conformations defined by Molprobity

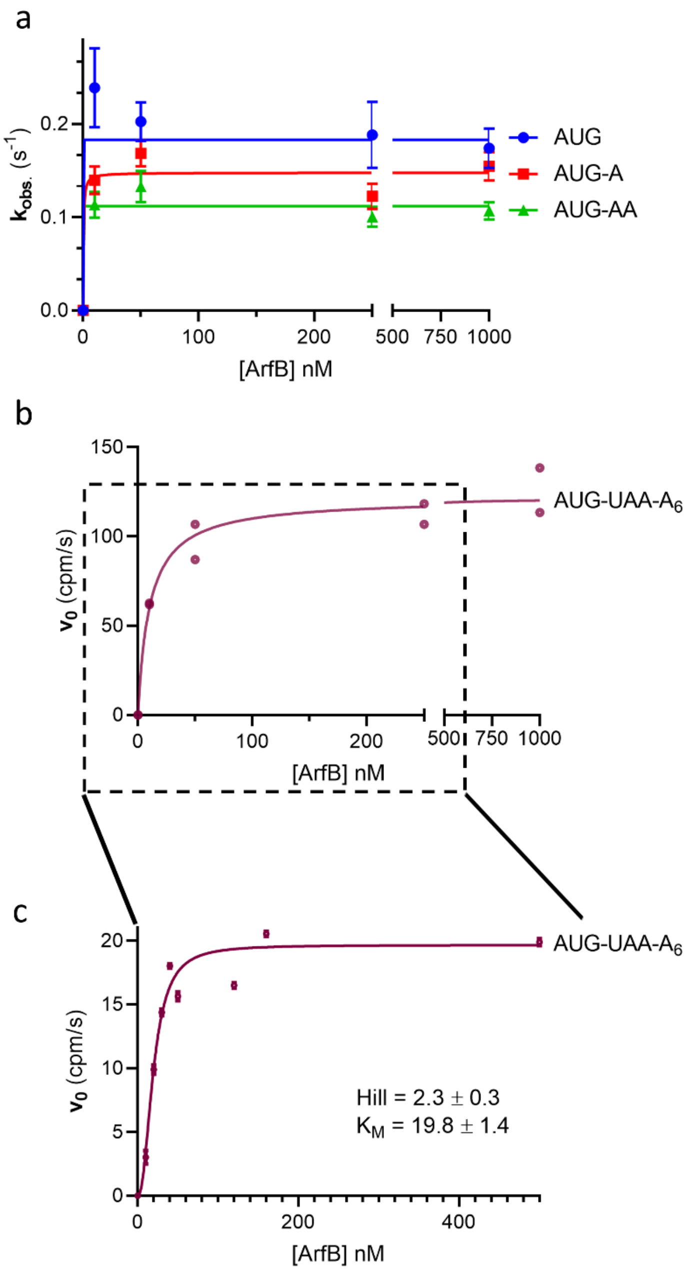

Supplementary Figure 1. Catalytic efficiencies of ArfB on short and +9 mRNAs. (a-b) Catalytic rates for ArfB (10-1000 nM) mediating fMet release from 10 nM 70S complexes formed on short (a) and +9 (b) mRNAs (error bars in panel a represent errors of fit obtained from two independent time progress curves with at least 7 time points each). On short mRNAs, the K_M is estimated at ⩽ 10 nM, comparable to those for release factors 1 and 2 measured under similar conditions [1,2]. (c) Fitting of the Michaelis-Menten curve for ArfB-mediated fMet release from the +9 complex results in an estimated K_M of 19.8±1.4 nM and Hill coefficient of 2.3±0.3 (errors of fit were obtained from two independent time progress curves with at least 7 time points each). These experiments were done as described in Methods, with the following modification to match the conditions for cryo-EM samples: magnesium acetate concentration was 20 mM, buffer pH was 7, experiments were conducted at room temperature.

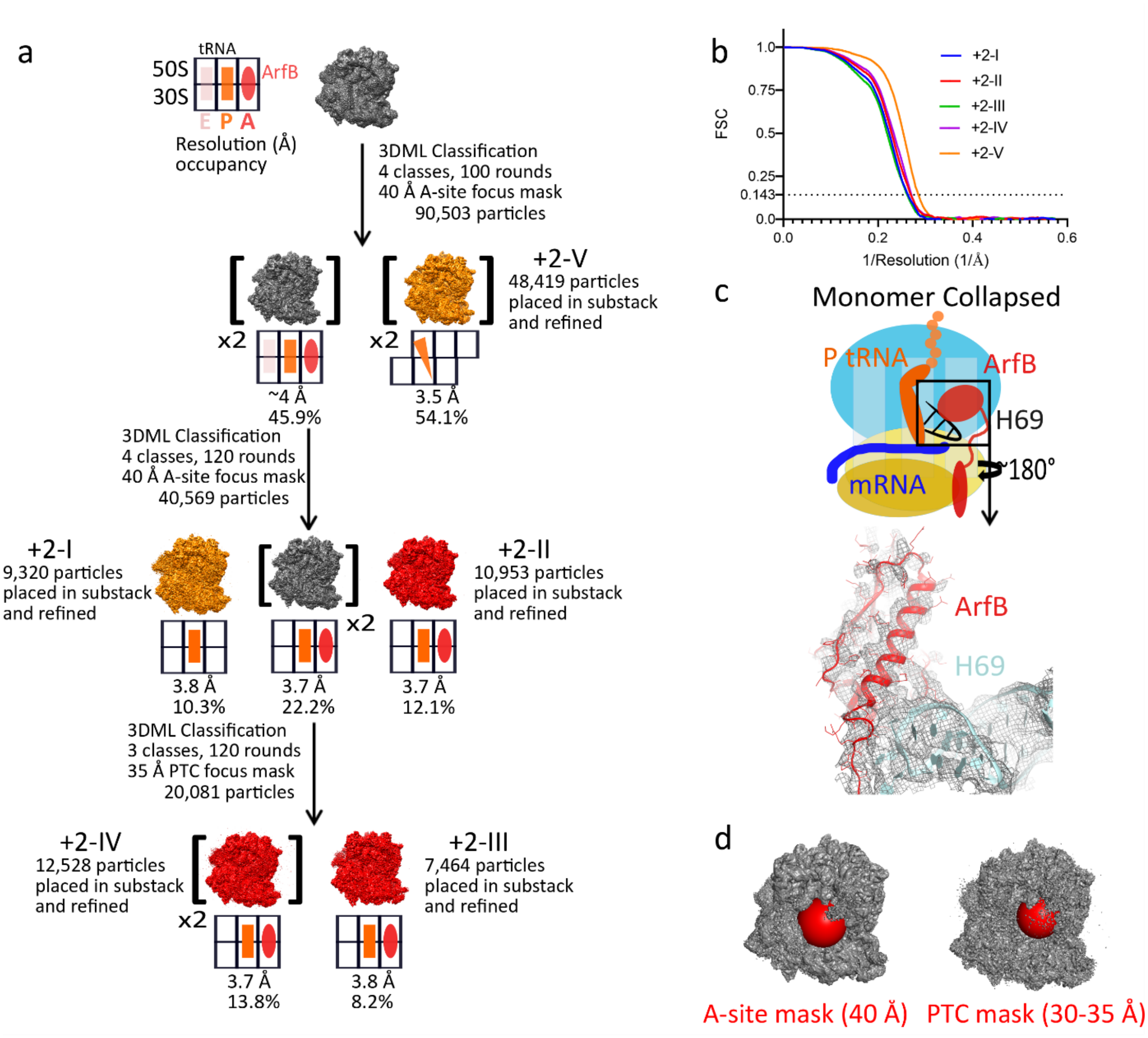

Supplementary Figure 2: Cryo-EM data classification for the +2 mRNA complex. (a) Scheme of the maximum-likelihood classification strategy to obtain the final maps and state occupancies. Maps used in structural modeling are red (with ArfB) and orange (no ArfB); all other maps are in grey. (b) Fourier shell correlation (FSC) between even- and odd-particle half maps for Structures +2-I through +2-V. (c) Cryo-EM density (mesh; σ=2.7) showing the long helix of the N-terminal domain of ArfB collapsed on H69 (Structure +2-IV). Map was sharpened to B= -25 $Å^2$. (d) Positions of the A-site and PTC masks (red spheres) used for particle classification.

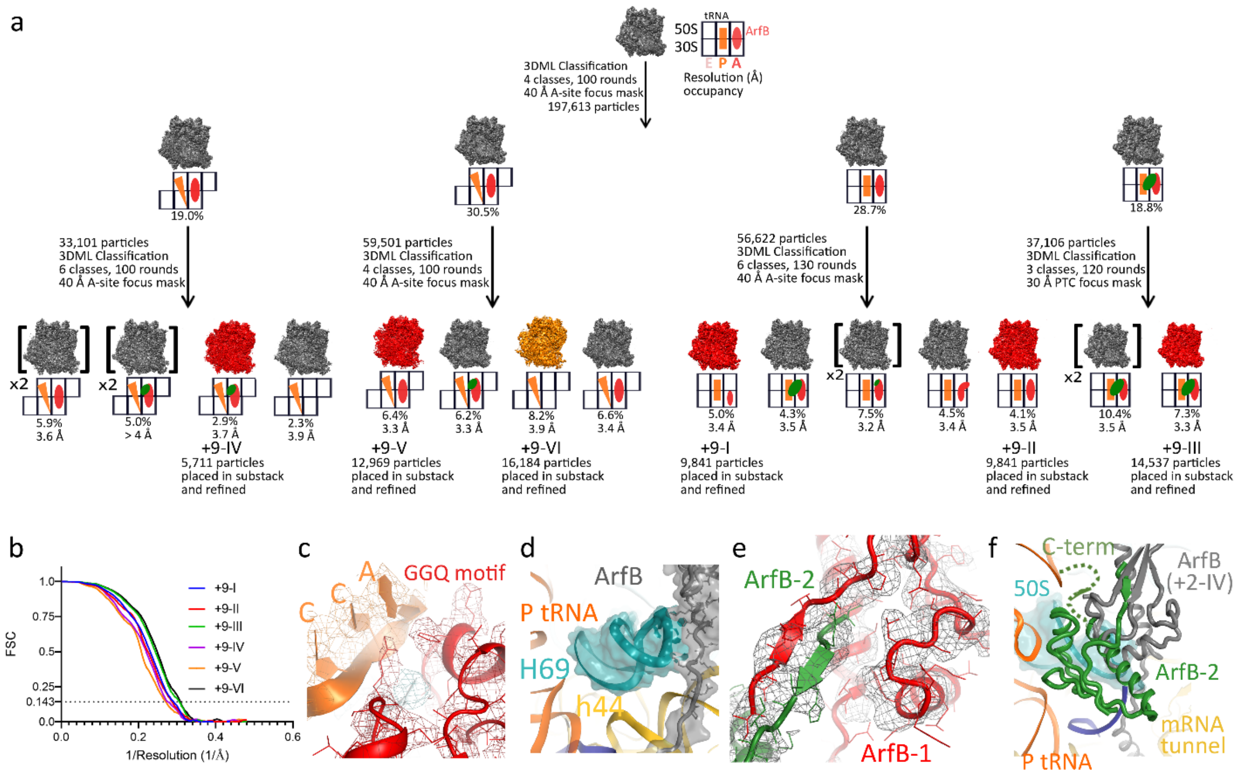

Supplementary Figure 3. Cryo-EM data classification for the +9 mRNA complex. (a) Scheme of the maximum-likelihood classification strategy to obtain the final maps and state occupancies (using masks shown in Supplementary Fig. S2d). Maps used in structural modeling are red (with ArfB) and orange (no ArfB); all other maps are in grey. (b) Fourier shell correlation (FSC) between even- and odd-particle half maps for Structures +9-I through +9-VI. (c) Cryo-EM density (mesh; ArfB σ= 4.2, 23S and P tRNA σ=5) showing the catalytic region of ArfB (red) next to the CCA end of P-site tRNA (orange) in the peptidyl transferase center (Structure +9-III). Map was sharpened with a B= -25 Å^2. (d) Superposition of Structure +9-VI with Structure +9-IV showing that H69 (cyan) detached from h44 (yellow) would sterically clash with the linker of ArfB-1 (grey). Superposition was achieved by aligning 16S rRNA. (e) Cryo-EM density for the interaction between N-terminal domains of ArfB-1 and ArfB-2 (mesh; σ=4; Structure +9-III is shown). (f) Superposition of collapsed ArfB from +2-IV onto ArfB-2 from +9-III shows different positions of ArfB molecules and ArfB-2 C-terminus (dotted green line) being incompatible with binding in the mRNA tunnel. Superposition was achieved by aligning 23S rRNA.

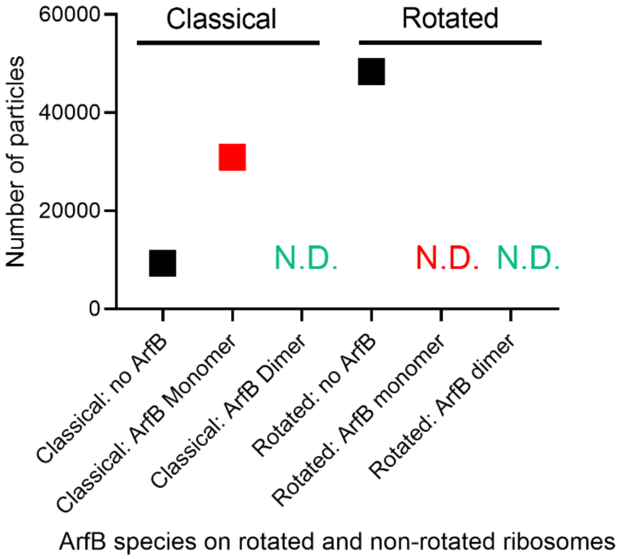

Supplementary Figure 4. Particle distribution of ArfB on rotated and non-rotated 70S ribosomes in the +2 mRNA complex. Monomer was defined as either N or C domain density with no evidence of ArfB-2. Dimer was defined as monomer plus any component of ArfB-2. N.D. –not detected. N= 1 cryo-EM data set (90,503 particles).

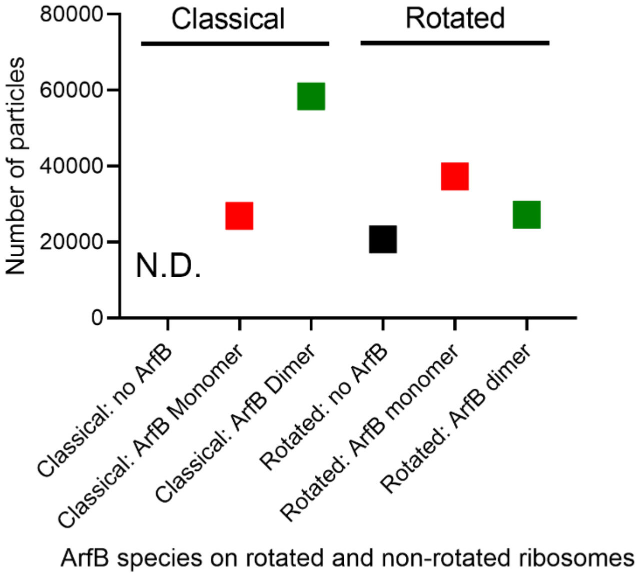

Supplementary Figure 5. Particle distribution of ArfB monomers and dimers on rotated and non-rotated 70S ribosomes in the +9 mRNA complex. Monomer was defined as either N or C domain density with no evidence of ArfB-2. Dimer was defined as monomer plus any component of ArfB-2. N.D. –not detected. N= 1 cryo-EM data set (197,613 particles).

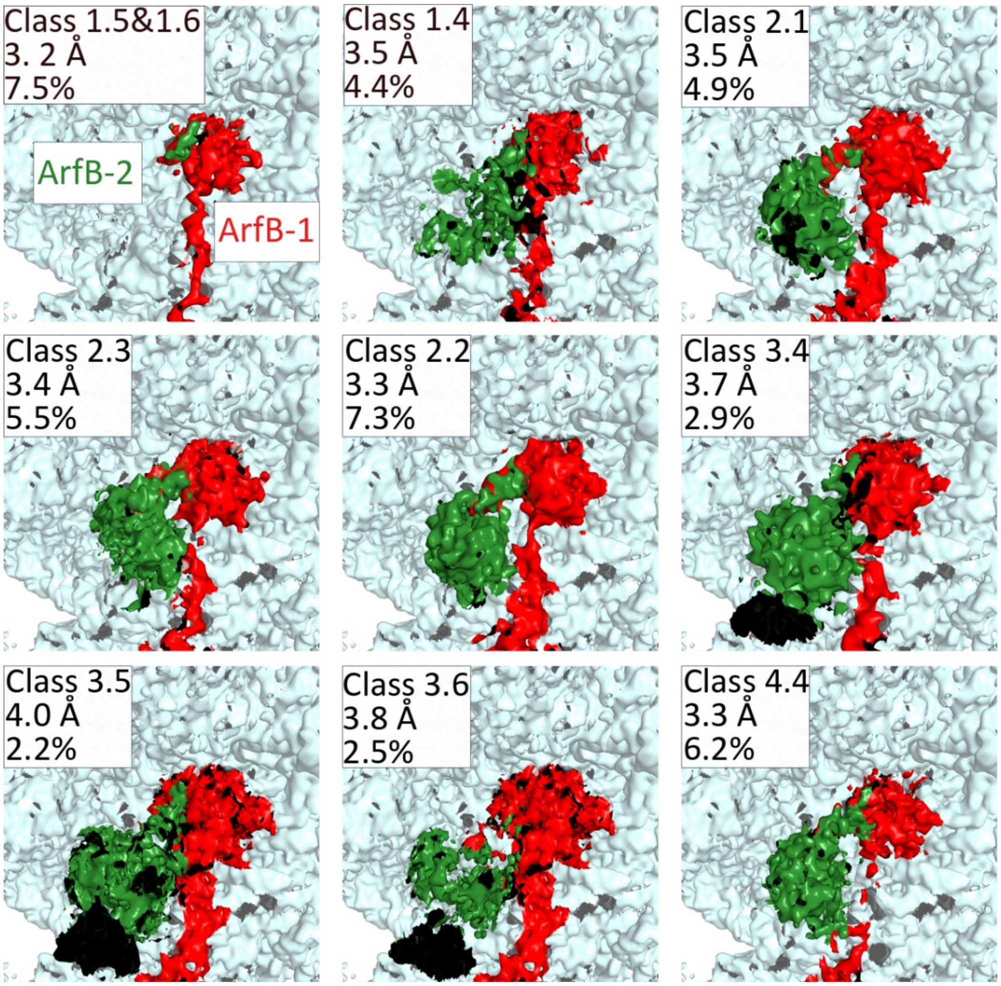

Supplementary Figure 6. Cryo-EM maps (surface) with dimeric ArfB consistent with dynamics of ArfB-2 (green) relative to ArfB-1 (red) in the +9 mRNA complex.

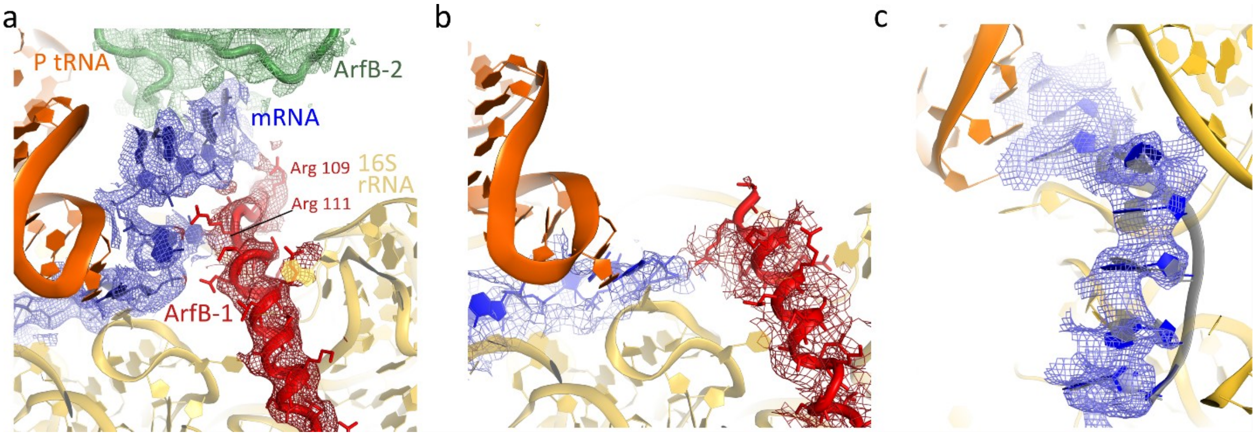

Supplementary Figure 7. Cryo-EM density for mRNA and/or ArfB-1 C-terminal tail on non-rotated and rotated ribosomes. (a) Both mRNA (blue) and ArfB-1 C-terminal tail (red) are well resolved on non-rotated ribosomes in structure +9-**III** (B= -25 Å^2; σ=1.8). (b) mRNA overhang and C-terminal tail are poorly resolved on rotated ribosomes in structure +9-V (B-sharpening not applied; σ=3.0). (c) mRNA (blue) resides in the mRNA tunnel of the rotated structure without ArfB (+9-VI). High-resolution 70S crystal structure with mRNA modeled in the mRNA tunnel is shown in gray for reference (B=50 Å^2; σ=1.7) [3](PDB 4V6G).

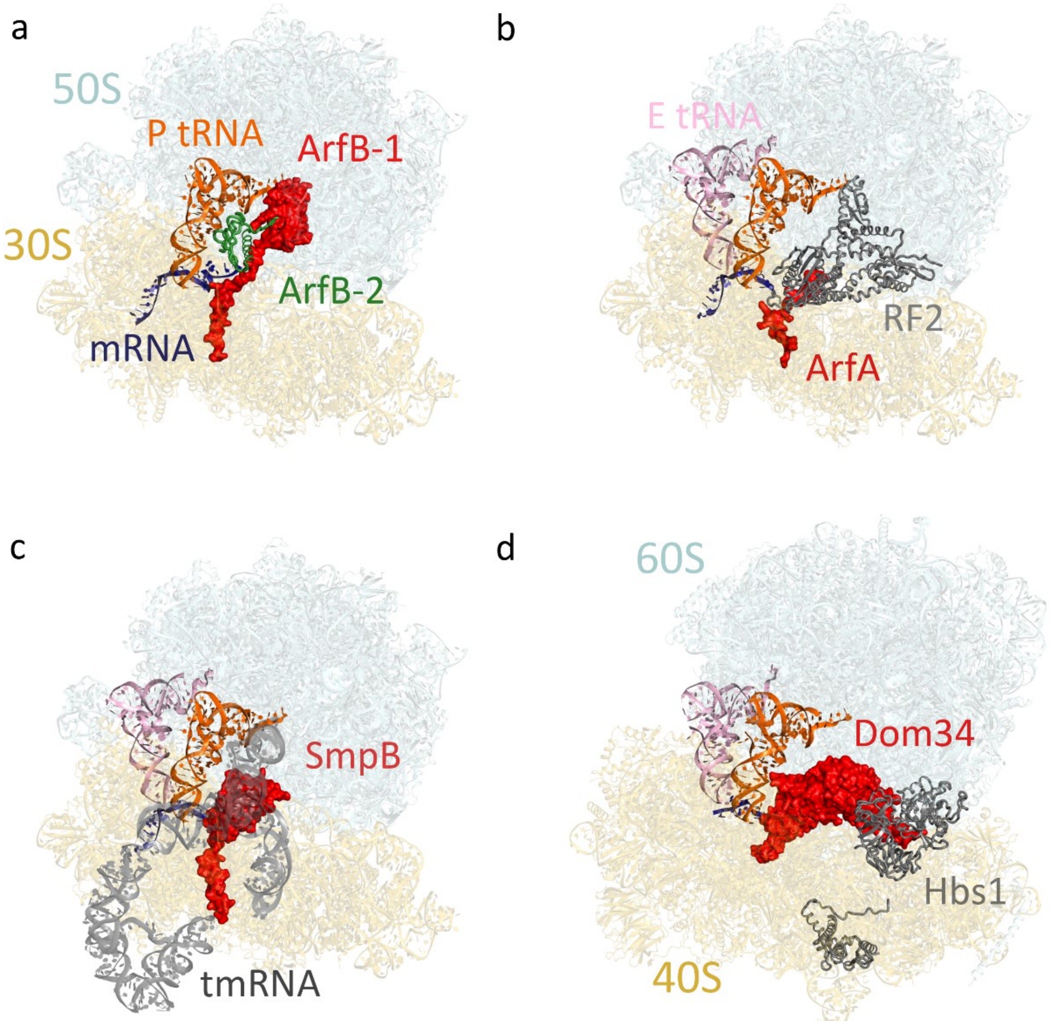

Supplementary Figure 8. Bacterial and eukaryotic ribosome rescue pathways. (a-c) Cryo-EM structures of bacterial rescue factors (red, green and gray) that bind in the mRNA tunnel of 70S ribosomes (pale yellow and cyan)—ArfB (a), ArfA (b), and SmpB (c). ArfB is bound to the ribosome programmed with +9 mRNA (blue; this work) while ArfA•RF2 and SmpB•tmRNA trans-translation complex were captured on ribosomes programmed with mRNA without an overhang (PDB: 5U9F and 6Q97). (d) Dom34•Hbs1 (green and red) bound to an 80S ribosome (pale yellow and cyan) programmed with mRNA without an overhang (PDB: 5LZY).

References

1 Freistroffer, D. V., Kwiatkowski, M., Buckingham, R. H. & Ehrenberg, M. The accuracy of codon recognition by polypeptide release factors. *Proc Natl Acad Sci U S A* **97**, 2046-2051, doi:10.1073/pnas.030541097 (2000).

2 Svidritskiy, E. & Korostelev, A. A. Conformational Control of Translation Termination on the 70S Ribosome. *Structure* **26**, 821-828.e823, doi:10.1016/j.str.2018.04.001 (2018).

3 Jenner, L., Demeshkina, N., Yusupova, G. & Yusupov, M. Structural rearrangements of the ribosome at the tRNA proofreading step. *Nature structural & molecular biology* **17**, 1072-1078, doi:10.1038/nsmb.1880 (2010).
